# Frustration Tolerance and Personality Traits in Patients With Substance Use Disorders

**DOI:** 10.3389/fpsyt.2019.00421

**Published:** 2019-06-14

**Authors:** David Ramirez-Castillo, Carlos Garcia-Roda, Francisco Guell, Javier Fernandez-Montalvo, Javier Bernacer, Ignacio Morón

**Affiliations:** ^1^Mind-Brain Group, Institute for Culture and Society, University of Navarra, Pamplona, Spain; ^2^Faculty of Education and Psychology, University of Navarra, Pamplona, Spain; ^3^Department of Health Sciences, Public University of Navarra, Pamplona, Spain; ^4^Department of Psychobiology and Research Center for Mind, Brain, and Behavior (CIMCYC), University of Granada, Granada, Spain

**Keywords:** substance addiction, frustration tolerance, ambulatory treatment, MCMI-III, therapeutic community

## Abstract

Previous research has suggested the prevalence of certain personality traits, some of which are related to a disorganized attachment, in substance abuse disorders. Further, frustration tolerance (FT) has been proposed as an important factor in addiction, both at the inception—following the “self-medication” hypothesis—and regarding treatment compliance. In turn, an inadequate response to frustrating events has been also associated with a disrupted attachment. Our goal is to explore the mediational role of FT in the relationship between personality traits and two different treatments for substance addiction: therapeutic community (TC) and ambulatory treatment (AT). Eighty-four subjects with substance abuse disorder were recruited in total (22 female), including 46 volunteers (13 female) in TC and 38 (9 female) in AT. They were assessed with Rosenzweig’s test for FT and the Millon Clinical Multiaxial Inventory-III (MCMI-III) test to evaluate personality factors. By comparing with a control sample (335 volunteers, 268 female), we found that FT was lower in patients. Between therapeutic groups, FT was significantly lower in TC. Depressive, antisocial, sadistic, negativistic, schizotypal, borderline, paranoid, anxiety, dysthymia, alcohol use, drug use, posttraumatic stress disorder (PTSD), thought disorder, and delusional disorder traits were suggestive of pathology in the clinical samples and were significantly different between control, AT, and TC groups. Further, anxiety and PTSD traits were higher in TC than in AT. A mediational analysis revealed that the effect of anxiety and PTSD scales on therapeutic group was partially mediated by FT. In conclusion, FT and its interplay with personality traits commonly related to disorganized attachment (anxiety and PTSD) might be important factors to consider within therapeutic programs for persons with substance addiction.

## Introduction

Drug addiction withdrawal implies a set of physiological and psychological challenges that should be faced by the patient and taken into account by the therapist (1–3). Physiological alterations appear a few hours after the cessation of drug administration, do not extend longer than 3 weeks (1), and can be alleviated by pharmacological treatment (4). However, psychological challenges are more persistent (5), and pharmacological intervention is not always effective to treat them (6, 7). Frustration is one of the common negative emotions involved in withdrawal (8–18), and its experience during detoxification significantly contributes to relapse and treatment discontinuation (7). Moreover, following the self-medication hypothesis, it has been proposed that illicit-drug consumption—and subsequent addiction—could be used as a means of alleviating negative emotions such as frustration (19).

Taking this into account, the ability of the patient to tolerate frustrating events might be an important factor in substance addiction, both in the development of the disorder and during treatment. Frustration is defined as a negative emotional response triggered after the omission and/or devaluation of an expected reward (20). Animal research has extensively demonstrated the influence of this negative emotion on substance abuse [see, for example, Ref. (21)]. Concerning humans, frustration tolerance (FT) is negatively associated with the number of relapses (22) and positively predicts recovery from alcoholism (23, 24). It is also an essential component of the complex construct of distress tolerance (25), which is, in turn, an important factor of withdrawal (26–28).

Previous studies have suggested the attachment hypothesis of addiction as an alternative to self-medication. Attachment is defined in developmental psychology as the emotional tie that connects the child with his or her main caregiver (29, 30). In her first works, Mary Ainsworth described three main types of attachment, namely, secure, anxious insecure, and avoidant insecure. Nowadays, the most common classification includes organized (secure, insecure–avoidant, or insecure–resistant) and insecure–disorganized attachment types (31). Within organized attachment, insecure children are hesitant to rely on their main caregiver in distress situations, due to a prior unreliable response from the adult. They are considered organized because children can develop strategies to handle stressful situations. In contrast, children with a disorganized attachment show a fearful, conflicted, or apprehensive behavior when reunited with their caregivers after distress (32). It is important to note that a disorganized attachment may co-exist with any of the organized subtypes, mostly insecure (33). In adults, insecure attachment is mostly manifested in enhanced avoidant and anxious behaviors. Like children, adults expect unresponsive peers in stressful situations, leading them to avoid close relationships or to desire proximity but lacking trust in their partners (32). Current research has proposed that an insecure (disorganized) attachment may be a risk factor for developing substance addiction (34–36). Moreover, a disorganized mother–child attachment can be associated with poorer FT in children (37, 38), and adults with insecure attachment are prone to show problems coping with emotion regulation (39, 40). Therefore, since FT is a relevant factor for both the self-medication hypothesis of addiction and disorganized attachment (which in turn may be a risk factor for developing substance addiction), the assessment of FT in patients with substance use disorders may help bring together both fields of research.

Interestingly, other authors have highlighted the influence of personality traits on addiction and attachment. Magor-Blatch and collaborators (41) explored pathological symptoms and clinical syndromes, assessed by the Millon Clinical Multiaxial Inventory-III (MCMI-III) (42), in a large sample of amphetamine-type substance users. They found scores in the pathological range in anxiety, bipolar, borderline, dependent, narcissistic, negativistic, and sadistic scales, although these values did not predict completion of the therapeutic program. Fernandez-Montalvo et al. (43) found that over 76% of patients in therapeutic community (TC) had a pathological personality, antisocial being the most prevalent trait (43%). Other studies report a significant presence of antisocial, anxiety, depressive, borderline, bipolar, and posttraumatic stress disorder (PTSD) traits in patients with substance dependency (44–47). Psychopathology is understood under the scope of attachment theory as an adaptive resource to compensate for an insecure attachment (48). Previous research has demonstrated a relationship between certain elements of a dysfunctional attachment (unresolved loss and unresolved trauma) and personality (49). Using the Adult Attachment Interview, these authors found that borderline and anxiety traits were higher in trauma inpatients with unresolved attachment. Furthermore, within the scope of the self-medication hypothesis of addiction, antisocial personality was described as a mediator of the relationship between alcohol consumption and parental bonds in male college students (50). Besides, other authors found a relationship between anxiety and insecure attachment styles in alcohol-addicted inpatients: anxiety traits were significantly higher in participants with insecure, compared with secure, attachment (51). Beyond the scope of addictive disorders, a disorganized attachment has been associated with higher values of paranoid, borderline, and histrionic traits in adults (52).

The present study intends to contribute to this field of research by evaluating personality traits and FT in subjects included in two different therapeutic approaches: TC and ambulatory treatment (AT). To the best of our knowledge, our research is the first to explore FT and personality traits in both types of treatment, since previous works have focused on TCs (11, 14, 22, 41, 53–55). Although we do not measure attachment directly, we aim to evaluate the association between the type of withdrawal program (TC and AT) (which is an indirect indicator of addiction severity and risk of social exclusion) (56), the presence of pathological personality traits that have been previously related to a disorganized attachment (i.e., antisocial, anxiety, PTSD, borderline) [see, for example, Ref. (57)], and the role of FT as a mediating factor between them.

Considering this, the objectives of our research are: 1) to evaluate FT in TC and AT groups; 2) to explore the personality traits of the whole clinical sample under study; 3) to compare personality traits between therapeutic groups, focusing on those previously related to disorganized attachment; and 4) to explore whether the influence of personality on treatment group is mediated by FT. A deeper knowledge of negative psychological emotions involved in withdrawal, such as frustration, and its mediation in the relationship between personality and treatment, may help improve these therapeutic programs and their probability of success.

## Methods

### Participants

Participants under treatment for substance abuse were recruited through an advertisement made by the therapists of Asociación Proyecto Hombre in the centers of Navarra and Granada. All volunteers signed an informed consent, and the protocol was approved by the Committee for Ethics in Research of the University of Navarra. Data were collected between 2015 and 2016. Proyecto Hombre verified, by means of urine tests, that participants were not consuming any drug—other than tobacco—during the 2 weeks prior to their psychological assessment. The total number of participants was 84 patients (22 female) from both centers. Age ranged from 20 to 63 years (40.06 ± 1.10, mean ± standard error of the mean), and it was not significantly different between male (39.31 ± 1.17) and female (42.18 ± 2.58) participants: Student’s *t*(82) = −1.156, *p* = 0.251. Concerning the therapeutic program, 46 participants (13 female) were in TC, whereas 38 received AT (9 female). The mean age of these groups was also similar: age(TC) = 38.70 ± 1.54, age(AT) = 41.71 ± 1.53; *t*(82) = −1.377, *p* = 0.172. A chi-square test of independence showed that gender composition was not different between therapeutic groups (χ^2^(1) = 0.225, *p* = 0.635). At the time of assessment, the duration of treatment was similar in both groups: TC, 4.12 ± 0.38 months; AT, 4.80 ± 0.34 months, *t*(82) = 1.317, *p* = 0.192. We did not collect information about psychopharmacological medication prescribed to volunteers (**Table 1**).

**Table 1:** Characteristics of the clinical and control samples included in the study.

	Clinical			Control		Matched	
	TC	AT	Whole	Whole	MCMI	Clinical	Control
N	46	38	84	335	76	29	26
Age	38.7 ± 1.54 (20–60)	41.7 ± 1.53 (24–63)	40.1 ± 1.1 (20–63)	21.1 ± 0.32 (18–58)	23.2 ± 1.21 (18–58)	35.6 ± 2.16 (20–56)	36.3 ± 2.37 (20–58)
Gender							
Female	13	9	22	268	59	21	20
Male	33	29	62	67	17	8	6
Treatment duration (months)	4.1 ± 0.38	4.8 ± 0.34	4.4 ± 0.04				

Descriptive statistics are means ± standard errors of the mean. In parentheses, age range. “Matched” refers to samples randomly matched by MedCalc software (see Methods for details).

AT, ambulatory treatment; MCMI, subsample of control participants evaluated with the MCMI-III (see Methods for details); TC, therapeutic community.

Inclusion criteria for the participants were: i) fulfilling the diagnostic criteria of the Diagnostic and Statistical Manual of Mental Disorders, 4th Edition, Text Revision (DSM-IV-TR) for substance dependence, substance abuse, and substance withdrawal; ii) participation in TC or AT for at least 2 weeks before data collection; and iii) presenting physical or psychological harm and/or dependence due to substance consumption in the past.

In order to assess FT in substance abuse with respect to the general population, we recruited a control sample (*N* = 335, 268 female), which included community controls (next of kin of the patients) and university students. Exclusion criteria were: 1) alcohol or substance abuse or dependence, as assessed by the Alcohol Use Disorders Identification Test (AUDIT) and Alcohol, Smoking and Substance Involvement Screening Test (ASSIST) tests; 2) self-reported previous treatment for substance abuse dependence; and 3) self-reported history of neurological or mental disorders. All control participants signed the informed consent to take part in the study. This sample was not matched in terms of sex and age with the clinical group. Thus, in the Results section, we present three different strategies to compare FT between both samples. In addition, a subsample of 76 participants from the initial control sample was assessed with the MCMI-III questionnaire (see below and **Table 1** for details).

#### Procedure and Therapeutic Strategies

Asociación Proyecto Hombre is a Spanish institution for treating and preventing addiction, involving 27 centers and 16,600 persons under treatment annually. They work in three fundamental areas: prevention, rehabilitation, and reintegration of former users (www.proyectohombre.es). The tests were conducted in the centers of Proyecto Hombre in the Spanish provinces of Granada and Navarra. Among others, they offer TC and AT therapeutic strategies. The former is inpatient, and the latter is outpatient, involving 1.5–2 h of treatment 5 days a week (Monday to Friday). Patients are assigned to either program depending on their preferences, as well as on the therapists’ recommendation based on their social situation. It is important to note that the assignment to TC points to a more compromised social and/or pathological situation, since inclusion in TC is related to the fact that patients do not or cannot have employment, or they do not have enough social support to tackle withdrawal treatment by themselves (56). The most common profile of AT patients, however, is a jobholder supported by family and acquaintances. In spite of this, therapeutic strategy is the same in both programs: group therapy for psychological and emotional aspects such as problem solving, anxiety control, emotional regulation, or relapse prevention, for instance. The only difference, apart from the number of hours spent daily in the program, is the emphasis on achieving personal and social autonomy in TC. Although the total time spent in the program depends on each person, TC treatment usually lasts 6–10 months, and AT is maintained for at least 1 year. Participants were allowed to smoke tobacco before assessment, so nicotine withdrawal is not expected to influence the study.

All data were anonymized and coded for each participant. First, the MCMI-III test was administered for approximately 45 min. Then, subjects were evaluated with the Rosenzweig test for about 25 min. After a short break in the same room, participants were screened for substance use, abuse, dependency, and damage. This procedure was also followed by the control sample, although the MCMI-III test was administered only to a subsample of 76 participants, including university students and community controls.

#### Questionnaires

*Millon Clinical Multiaxial Inventory-III (MCMI-III)*. This test (42) is used for testing clinical personality patterns (11 scales), severe personality pathology (3 scales), clinical syndromes (7 scales), and severe clinical syndromes (3 scales). It consists of 175 true/false items, and it also contains 3 control scales. This inventory identifies personality features underlying symptoms, and it is commonly used to assist clinicians in diagnosis and for therapy selection. According to the standards (58), scores between 60 and 74 are suggestive of symptoms at a subclinical level, values of 75–85 indicate presence and prevalence of the pathology or syndrome, and scores over 85 point to prominence of the pathology or syndrome.

*Rosenzweig Picture Frustration Test, PFT*. This is a semi-structured projective test to assess tolerance or intolerance to a frustrating situation (59). It consists of 24 vignettes where pairs of characters are interacting. The subject assumes the role of one of the characters and provides his or her expected behavior in that situation. The test provides an FT index that is calculated through a simple procedure: the response for each vignette is scored by two different raters (from 0 to 2) considering the degree of aggression, avoidance, blocking, or coping (2 = aggressive response, 0 = non-emotional response). In our study, inter-rater reliability of this assessment was very high (average intraclass correlation coefficient = 0.943). In spite of the high reliability, the final score of each vignette was agreed upon in case of a mismatch between the raters. Higher scores indicate lower FT (higher level of aggressive response).

*Substance use, abuse, and dependence*. All participants were screened with a 13-item test to identify the substances they used (in the case of patients, before joining the therapeutic program) (alcohol, ecstasy, heroin, speed, cocaine, caffeine, tobacco, cannabis, hallucinogens, tranquilizers, and marijuana). The two last items indicated substance dependency and physical or psychological harm due to substance consumption. This test is based on the ASSIST 3.0, published by the World Health Organization (WHO) (60).

Subjects were also screened with the AUDIT (WHO) in its Spanish version (61).

## Statistical analysis

All statistical analyses were performed in Stata 12.1. We present three different strategies to compare FT between both samples: 1) unpaired *t* test including all subjects (clinical vs. control sample); 2) multiple regression to predict FT (dependent variable) from group (case = 1, control = 0), controlling by age and sex (male = 1, female = 0); and 3) randomly choosing age- and sex-matched subsamples of both groups with MedCalc (Ostend, Belgium) and then comparing FT with an unpaired *t* test. In order to achieve matching, the software randomly selected the maximum number of cases within group that produced non-significant differences between groups for age and sex. Therefore, matches were not necessarily exact. A Shapiro–Wilk test was conducted to assess normality. For normally distributed samples, means and standard errors of the mean are reported; for non-normal distributions, we report medians and interquartile ranges (IQRs). Comparisons between therapeutic groups were carried out with parametric *t* tests, due to sample sizes larger than 30. A multivariate analysis of variance (MANOVA) was used to test the omnibus differences in personality traits between therapeutic groups. The effect of covariates (i.e., duration of treatment) was assessed with analysis of covariance (ANCOVA) and a MANOVA, respectively.

In order to assess the mediational role of FT on the influence between personality traits and therapeutic group, we performed mediational analyses as described by MacKinnon and Dwyer (62), and as explained by Kenny (63). We used the command binary_mediation available in Stata’s repositories. In our case, we wanted to test whether the relationship between certain personality traits (for example, anxiety) and the assignment to a particular therapeutic group (TC or AT) was influenced by FT. In short, the analysis explores first the total effect of the independent variable (i.e., anxiety personality trait) on the dependent variable (i.e., inclusion in either AT or TC), in this case with a logistic regression (AT = 0, TC = 1). Then, the total effect is divided into the direct effect (the influence of the independent variable, i.e., anxiety) on the dependent variable (AT or TC) controlling for the mediator (FT) and the indirect effect (the influence of the independent variable, i.e., anxiety) on the mediator (FT), and the effect of the mediator (FT) on the dependent variable (AT or TC); if the direct effect is negligible and the other tests are significant, the mediational role of the variable may be assumed. Practically, to fully assess the mediational role of a variable, four steps must be fulfilled: 1) the independent variable (i.e., anxiety) must have an effect on the dependent variable (treatment: TC = 1 or AT = 0); 2) the independent variable must have a significant effect on the mediational variable (FT as assessed by the PFT); 3) the mediational variable must have a significant effect on the dependent variable when controlling for the independent variable; and 4) the direct effect of the independent variable on the dependent variable, when controlling for the mediational variable, must be null (total mediation), or at least lower than when the mediational variable is considered (partial mediation).

Step 1 is assessed by a logistic regression and gives the total effect of the independent variable on the dependent variable (coefficient *c*). Step 2 is evaluated by a linear regression, taking FT as the dependent variable and the personality trait as predictor, and it provides coefficient *a*. Steps 3 and 4 are assessed with a logistic regression, including treatment as the dependent variable and the personality trait and FT as predictors. The influence of FT on treatment (controlling by personality trait) is coefficient *b* in the mediational model, and the effect of the personality trait on treatment (controlling for FT) is coefficient *c’*.

## Results

### Frustration Tolerance

**Figure 1:**
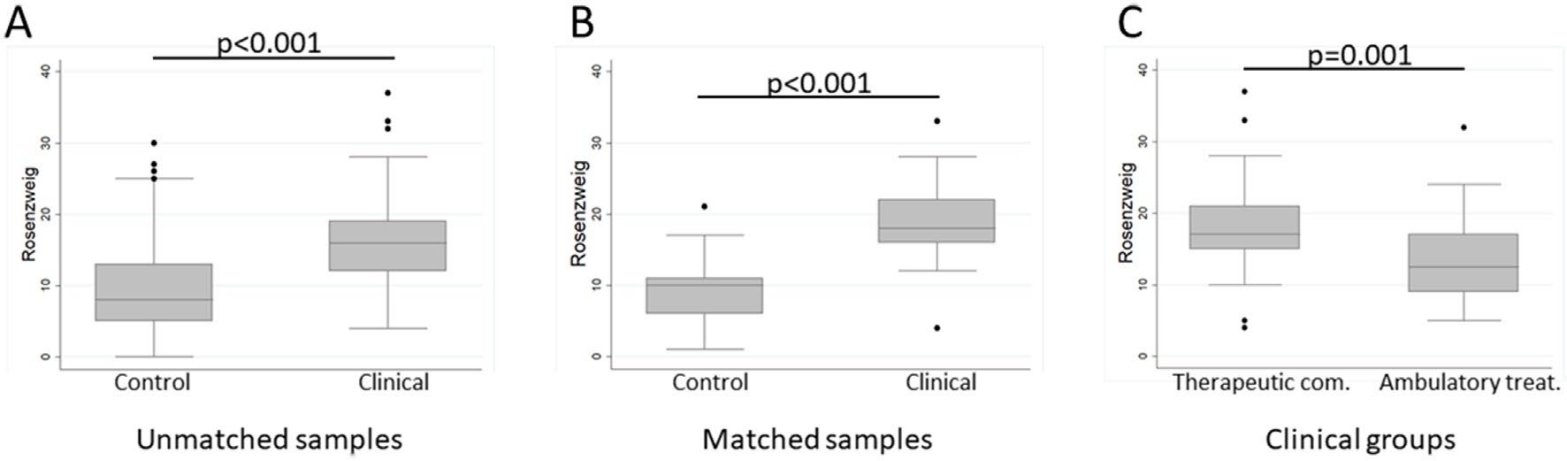
Box plots showing differences in frustration tolerance (FT) between clinical and control samples, as well as between therapeutic programs. **(A)** Rosenzweig Picture Frustration Test (PFT) scores in the unmatched clinical and control samples. **(B)**, median scores for the MedCalc-matched samples. **(C)** Rosenzweig PFT scores in therapeutic community and ambulatory treatment. Note that high values indicate poor FT.

Overall, the whole clinical sample (AT and TC) showed a score of 16 ± 6.5 (median ± IQR; Shapiro–Wilk *p* = 0.0235) in the Rosenzweig test (**Figure 1**). This is considered a medium FT according to the standards, as defined by Rosenzweig and based on the Dollard et al. theory of frustration–aggression (64), and points to an avoidant or blocking behavior when facing frustrating situations, masking a desire for aggression that the subject intentionally conceals (65). Note that higher scores on this test indicate a lower FT.

The control sample showed lower values on average (8 ± 8, Shapiro–Wilk *p* < 0.0001) (**Figure 1**), which reflect a strategic resolution of the frustrating vignettes based on social skills, empathy, and assertiveness. The comparison between these samples, unmatched for sex and age, yielded statistically significant results: *t*(417) = −9.99, *p* < 0.001, Cohen’s *d* = 1.22, pointing to a better FT in controls than in the clinical sample. In order to test whether this result was due to the difference in age (control, 21.09 ± 0.32 yr; clinical sample, 40.06 ± 1.1 yr) or sex composition (control, 80% female; clinical sample, 26.2%), we performed a linear regression to predict FT values from group, sex, and age. A significant regression equation was found [*F*(3,415) = 33.178, *p* < 0.001, η*p*² = 0.19], with a corrected *R* ^2^ = 0.188. The only significant regressor was group (*p*(group) < 0.001, *p*(sex) = 0.695, p(age) = 0.964), controlling by sex and age. Participants’ predicted FT was 9.18 + 7.10(group), where group was coded as 0 = control, 1 = clinical sample. These results were confirmed by randomly selecting subsamples for each group with MedCalc (*N*_control_ = 26, 20 female; *N*_clinical_ = 29, 21 female; age(control) = 36.31 ± 2.37; age(clinical) = 35.55 ± 2.16). In this case, we used non-parametric statistics since *N* < 30: Mann–Whitney *U* = 63.5, *z* = -5.295, *p* < 0.001, Cohen’s *d* = 1.815. In these subsamples, PFT was 10 ± 5 for controls and 18 ± 6 for the clinical sample (**Figure 1**).

In conclusion, as hypothesized, FT was lower in the clinical group than in the control sample, as measured by Rosenzweig’s PFT.

With respect to therapeutic groups, PFT values were higher in TC (18.22 ± 0.95, mean ± SEM; Shapiro–Wilk *p* = 0.0912) than in AT (13.34 ± 0.98; Shapiro–Wilk *p* = 0.0645) (**Figure 1**). As explained in the Methods section, these samples were matched for sex and age. A parametric *t* test confirmed statistical differences: *t*(82) = −3.54, *p* = 0.001, Cohen’s *d* = 0.78. Therefore, in accordance with our hypothesis, FT was lower in TC than in AT. Even though treatment duration was similar between groups, we performed an ANCOVA to confirm the difference between TC and AT in FT controlling for treatment duration (in months). As expected, between-group differences held when controlling by treatment duration at the time of assessment [*F*(1,25) = 1.83, *p* = 0.0302, η*p*² = 0.07].

### Personality Factors and Treatment Groups

We explored personality traits in TC and AT. Scores from the MCMI-III were processed as described by Millon et al. (58). All patients were assessed with this tool, whereas it was restricted to a subsample of 76 controls. Considering all the scales, Cronbach’s α was 0.90, showing a high internal consistency. With respect to individual subscales, values ranged from 0.9509 (borderline) to 0.9556 (narcissist).

In order to analyze the clinical relevance of each personality trait, we focused on those that were significantly different between groups (control, AT, and TC) and with a median equal to or above 60 for at least one of the clinical groups. Thus, we performed an ANOVA for each scale, using Bonferroni correction for multiple comparisons (*p* = 0.05/24 = 0.0021). Depressive, antisocial, sadistic, negativistic, schizotypal, borderline, paranoid, anxiety, dysthymia, alcohol use, drug use, PTSD, thought disorder, and delusional disorder traits fulfilled these criteria (**Table 2**). Due to the possible clinical relevance of these 14 scales, we focused on them for subsequent analysis.

**Table 2:** MCMI-III personality traits in the control, therapeutic community, and ambulatory treatment samples.

	Control (*N* = 76)	TC (*N* = 46)	AT (*N* = 38)	ANOVA *F*(2,157)	*p*
Clinical personality patterns					
Schizoid	26 ± 34	53.5 ± 16	51.5 ± 33	19.23	<0.0001*
Avoidant	32 ± 41	60 ± 30	51 ± 45	6.10	0.0028
Depressive	25 ± 27.5	61 ± 23	53 ± 41	21.84	<0.0001*
Dependent	42 ± 36	59.5 ± 24	53 ± 35	2.84	0.0616
Histrionic	68 ± 24.5	35 ± 36	47 ± 35	36.94	<0.0001*
Narcissist	63 ± 7.5	59.5 ± 21	64.5 ± 9	4.40	0.0138
Antisocial	60 ± 29	70 ± 13	69.5 ± 13	30.55	<0.0001*
Sadistic	45 ± 34.5	61.5 ± 16	64.5 ± 18	19.37	<0.0001*
Compulsive	63 ± 28.5	34 ± 32	44 ± 27	28.81	<0.0001*
Negativistic	44 ± 33	59.5 ± 14	61.5 ± 18	10.49	0.0001*
Masochistic	20 ± 32.5	54.5 ± 11	52.5 ± 30	24.77	<0.0001*
Severe personality pathology					
Schizotypal	24 ± 53	60 ± 8	48 ± 38	11.98	<0.0001*
Borderline	40 ± 43.5	65 ± 7	60 ± 31	21.53	<0.0001*
Paranoid	48 ± 47	63.5 ± 13	63.5 ± 19	11.63	<0.0001*
Clinical syndromes					
Anxiety	41.5 ± 58.5	82 ± 17	59 ± 49	13.42	<0.0001*
Somatoform	10 ± 27.5	46 ± 44	27.5 ± 42	7.71	0.0006*
Bipolar	60 ± 38.5	70.5 ± 19	63.5 ± 29	5.61	0.0044
Dysthymia	8 ± 29	64 ± 20	50 ± 46	38.83	<0.0001*
Alcohol use	60 ± 45.5	77.5 ± 16	74.5 ± 20	44.98	<0.0001*
Drug use	60 ± 33	89.5 ± 22	81 ± 25	74.58	<0.0001*
PTSD	30 ± 50	60 ± 10	38 ± 52	11.38	<0.0001*
Severe clinical syndromes					
Thought disorder	33 ± 43.5	69.5 ± 24	43 ± 44	24.15	<0.0001*
Major depression	12 ± 27	53.5 ± 36	33 ± 47	17.35	<0.0001*
Delusional dis.	60 ± 61	63.5 ± 10	64 ± 8	13.25	<0.0001*

Median ± interquartile range is reported for all variables, since most of the traits (82%) followed a non-normal distribution. In gray, personality traits with a median ≥60 for any of the clinical samples and significant differences between groups (control, AT, and TC).

*Significant differences between all three groups. Bonferroni correction is applied to determine critical p (0.05/24 = 0.0021).

AT, ambulatory treatment; TC, therapeutic community.

In order to assess whether these personality traits were different between therapeutic groups, we performed a MANOVA with the MCMI scales as dependent variables and treatment group as an independent variable. There was a statistically significant difference between treatment groups in MCMI scales, *F*(14, 69) = 2.06, *p* = 0.025, Wilks’ lambda = 0.705. As *post hoc* analyses, we performed two-tailed independent *t* tests for each variable, assuming a critical *p* value of 0.0036 (i.e., 0.05/14). Anxiety [*t*(82) = −3.42, *p* = 0.001; Hedges *g* = 0.743) and PTSD (*t*(82) = −3.23, *p* = 0.0018; Hedges *g* = 0.703) survived this threshold (**Table 3**).

**Table 3:** Between-group (clinical samples) differences in the MCMI-III personality traits suggestive of symptoms at a subclinical or clinical level.

	TC (*N* = 46)	AT (*N* = 38)	*t*	*p*
Clinical personality patterns				
Depressive	61 ± 23	53 ± 41	1.85	0.068
Antisocial	70 ± 13	69.5 ± 13	2.02	0.047
Sadistic	61.5 ± 16	64.5 ± 18	1.30	0.196
Negativistic	59.5 ± 14	61.5 ± 18	0.29	0.77
Severe personality pathology				
Schizotypal	60 ± 8	48 ± 38	2.33	0.022
Borderline	65 ± 7	60 ± 31	2.57	0.012
Paranoid	63.5 ± 13	63.5 ± 19	0.77	0.44
Clinical syndromes				
Anxiety	82 ± 17	59 ± 49	3.42	0.001*
Dysthymia	64 ± 20	50 ± 46	1.78	0.078
Alcohol use	77.5 ± 16	74.5 ± 20	2.31	0.023
Drug use	89.5 ± 22	81 ± 25	2.58	0.011
PTSD	60 ± 10	38 ± 52	3.23	0.0018*
Severe clinical syndromes				
Thought disorder	69.5 ± 24	43 ± 44	2.85	0.0055
Delusional dis.	63.5 ± 10	64 ± 8	0.49	0.622

Median ± interquartile range is reported.

*Significant differences between groups. Bonferroni correction is applied to determine critical p (0.05/14=0.0036).

AT, ambulatory treatment; TC, therapeutic community.

#### Influence of Personality Traits and Frustration Tolerance on Treatment Group

We hypothesized that FT may act as a mediational variable in the relationship between pathological personality traits and treatment group, which is an indicator of addiction severity. In order to test mediation, we followed the recommendations by Kenny (63), which assume the fulfillment of the four steps described in the Methods section of the present manuscript. We independently evaluated the effect of each scale suggestive of pathology (the 14 traits mentioned in the previous section) on treatment (TC = 1, AT = 0). Hence, we followed an approach suitable for dichotomous outcomes (66). Since we performed several different analyses, the critical *p* value was Bonferroni-corrected (*p* = 0.05/14 = 0.0036).

**Figure 2:**
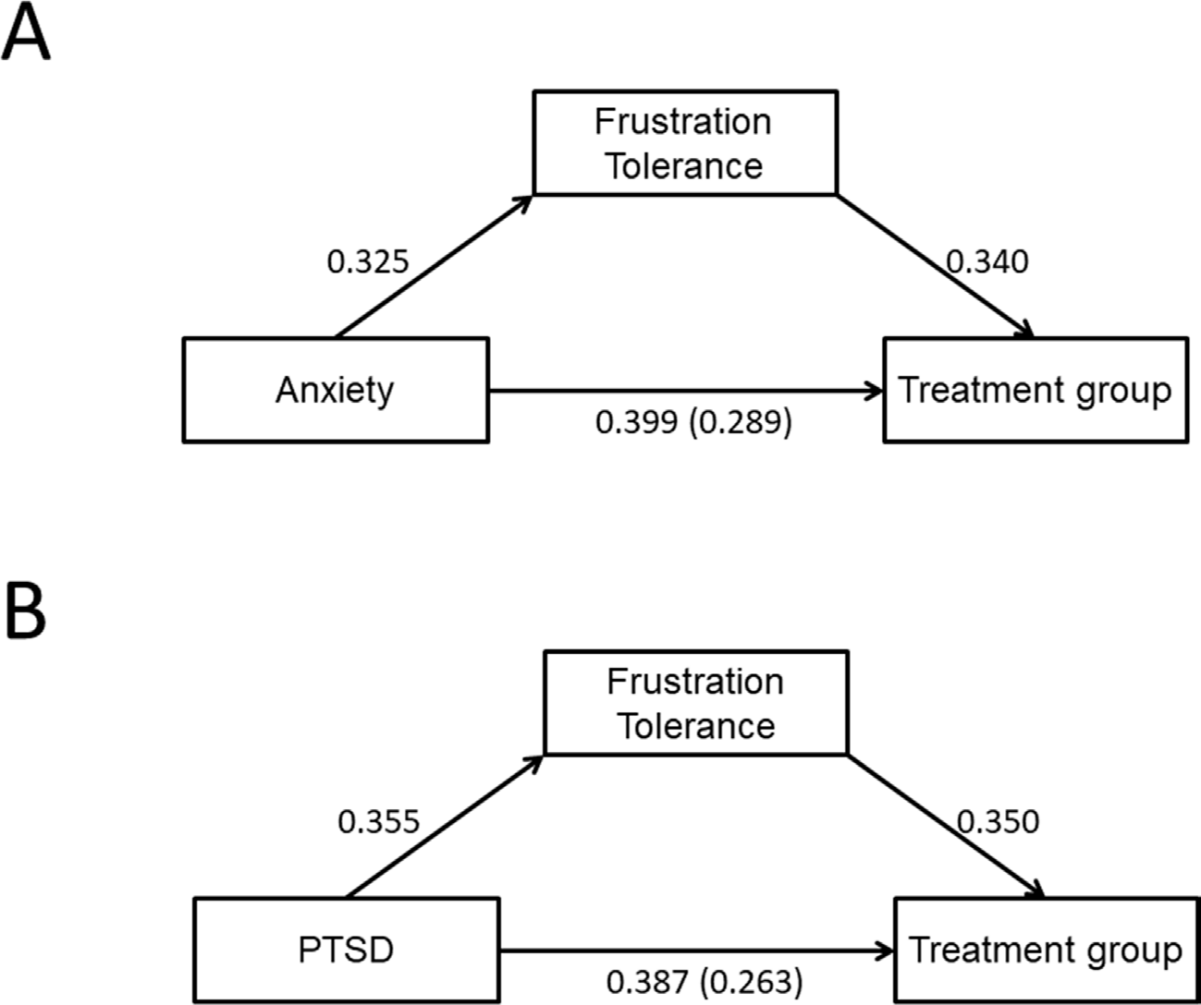
Mediation analyses between personality traits, FT, and treatment program (therapeutic community or ambulatory treatment). Personality traits are anxiety **(A)** and PTSD** (B)**. Numbers are standardized regression coefficients. Paths from each personality trait to FT correspond to coefficient *a*; paths from FT to treatment correspond to coefficient *b*; and paths between personality traits and treatment are total effects (*c*) (in parentheses, direct effect (*c’*) when controlling for FT).

As expected from the results of the previous section, only anxiety and PTSD survived Bonferroni correction when evaluating the influence of the independent variables (personality traits) on the dependent variable (treatment group) (Step 1): anxiety, χ^2^(1) = 10.91, *p* = 0.001; PTSD, χ^2^(1) = 9.80, *p* = 0.0017. These traits also influenced FT (Step 2), as assessed by a linear regression: anxiety, *F*(1,82) = 9.689, *p* = 0.003, η*p*² = 0.106; PTSD, *F*(1,82) = 11.84, *p* = 0.0009, η*p*² = 0.126. Both fulfilled Step 3, that is, PFT values significantly influenced treatment group when controlling for the corresponding personality trait (see standardized coefficients in **Table 4**): anxiety, *B*_PTF_ = 0.107 ± 0.044, *p* = 0.015; PTSD, *B*_PTF_ = 0.110 ± 0.044, *p* = 0.013. Finally, the influence of each personality trait on treatment group was reduced when controlling for FT, although they remained significant (Step 4; see **Table 4** for details): *B*_anxiety_ = 0.020 ± 0.009, *p* = 0.024; *B*_PTSD_ = 0.023 ± 0.011, *p* = 0.037. The significance of direct and indirect effects in each case was tested using bootstrapping procedures on standardized coefficients (5,000 samples), and the bias-corrected 95% confidence intervals are reported in **Table 3** (see also **Figure 2**).

**Table 4:** Mediational analyses between personality traits, frustration tolerance, and treatment group.

		Total effects (c)	Direct effects (c’)	Indirect effect (a)	Indirect effect (b)	Total indirect effects (a*b)	% Total effect mediated
Anxiety	β	0.399	0.289	0.325	0.340	0.111	27.7%
	BC CI 95%	0.155, 0.625	0.041, 0.542			0.033, 0.229	
PTSD	β	0.387	0.263	0.355	0.350	0.124	32.1%
	BC CI 95%	0.148, 0.602	0.040, 0.505			0.034, 0.259	

a (indirect effect), effect of the personality scale on frustration tolerance; b (indirect effect), effect of frustration tolerance on treatment group (1 = therapeutic community, 0 = ambulatory treatment), controlling for the corresponding personality trait; β, standardized coefficient; BC CI 95%, bias-corrected 95% confidence interval after bootstrapping standardized coefficients; c, total effect of personality scale on treatment group; c’, direct effect of personality trait on treatment type controlling for frustration tolerance.

In other words, an increase of one standard deviation in anxiety and PTSD personality traits increases the odds of receiving TC treatment to 1.49 and 1.47, respectively. Part of this effect (27.7% in the case of anxiety and 32.1% for PTSD) was mediated by FT, suggesting the presence of hidden mediators.

## Discussion

The present results show an association between personality traits, FT, and two different therapeutic strategies to overcome substance abuse: TC and AT. Concerning MCMI-III, there were scores suggestive of pathology and significant differences between the control and clinical samples in depressive, antisocial, sadistic, negativistic, schizotypal, borderline, paranoid, anxiety, dysthymia, alcohol use, drug use, PTSD, thought disorder, and delusional disorder scales. Regarding personality and addiction, a previous report (41) analyzed personality factors in TCs of substance users. They also found scores in the pathological range in drug and alcohol use, antisocial, sadistic, and anxiety traits, among others. Overall, MCMI-III scores were higher in their study, but the general organization of clinical personality and syndrome scales resembles our results. The globally lower scores of our participants may be due to the different nature of the samples included in both studies: whereas the present report included subjects addicted to alcohol, marijuana, cocaine, or benzodiazepines, that of Magor-Blatch et al. (41) was restricted to amphetamine-type prior users. Besides, we administered the MCMI-III test two weeks after the initiation of the program, whereas they assessed participants at the commencement of the therapy. Interestingly, the control sample assessed with the MCMI-III in our research showed relatively high values (≥60) in several subscales (histrionic, narcissist, antisocial, compulsive, bipolar, alcohol and drug use, and delusional disorder). Whereas antisocial, alcohol and drug use, and delusional disorder scores were significantly lower than in the clinical samples, the median value for histrionic and compulsive traits was significantly higher in control participants than in patients. The latter result is not surprising, since elevated scores in histrionic, narcissistic, and compulsive scales have been extensively reported for nonclinical groups, pointing to normal traits; moreover, they are associated with less severity of the disorder when present in clinical samples (67). With respect to the relatively high values in alcohol and substance use, the absence of dependence and harm indicators based on the AUDIT and ASSIST justifies the consideration of this sample as “control,” compared with the clinical samples.

One of the main goals of our research was to seek differences in personality factors between TC and AT groups. To our knowledge, our study is the first to compare personality patterns of substance users between both programs. Previous reports have focused on TCs (11, 14, 22, 41, 53–55, 68), while studies on outpatients addressed low-intensity drug abuse such as tobacco (69, 70), alcohol, or cannabis (17). In our study, focusing on those scales with a putative pathological meaning (with scores ≥60) that showed significant differences between the three groups, patients in TC showed higher scores than those in AT in all of the traits that were statistically different between both clinical samples (see **Table 3**). Patients in TC are more vulnerable due to the higher severity of their condition and/or a risk of social exclusion; in the case of Proyecto Hombre, the institution that hosted the volunteers included in our study, the main reason to be included in TC is insufficient family or social support. This, together with a severe substance addiction disorder, may pose difficulties for the patient to find or retain a job, stable housing, and consequently solid ground to recover from his or her condition. Due to this, patients in TC are at a higher risk for social exclusion. Our results confirm significant differences in a number of scales, pointing to an overall pathological condition that should be considered during treatment. The subscales that showed significant differences were anxiety and PTSD, with medium-large effect sizes (Hedges *g* values above 0.80 are considered large effects) (71). Previous research suggests usual co-morbidity of cocaine addiction with psychiatric disorders such as major depression, bipolar disorder, schizophrenia, PTSD, attention-deficit hyperactivity disorder (ADHD), anxiety, or borderline disorder (72). In fact, the European Monitoring Center for Drugs and Drug Addiction in 2015 stated that dual pathology and co-morbidity between drug addiction and mental disorders had risen up to 60%. According to our results, although we have not assessed co-morbidity per se, the higher scores in TC than in AT for most of the personality traits point to an enhanced vulnerability of patients in the former to develop mental disorders. This highlights the potential importance of a thorough personality profile before assigning a patient to either therapeutic program, as other authors suggest (6, 7).

With respect to negative emotional symptoms, our results show a lower FT in the clinical sample than in controls, and in TC compared with AT. The effect sizes of these results are quite large, indicating that about 96% of the matched control sample have better FT (lower scores) than the clinical sample, and about 79% of the TC sample have lower FT than the AT sample. Hence, this construct may be an important distinctive factor in substance abuse. Different stages of addiction, and relapse in particular, have been understood as a behavioral response to overcome the negative emotions (dysphoria, anxiety, irritability, etc.) that occur after the cessation of drug administration (73). Our result on the difference in FT between patients and controls may point to a crucial role in the development of addiction itself: in the presence of frustrating situations, when negative emotions are linked to a response of the pituitary–hypothalamus–adrenal axis (74), a poorer management of frustration may lead to its alleviation through the euphoric effects of recreational drugs. As has been recently suggested in children, negative emotions such as frustration may be regulated by the lateral prefrontal cortex (75). Moreover, a poor FT may trigger treatment discontinuation or relapse during withdrawal (22). We show here that patients in TC, where more frustrating situations are expected to occur due to the radical change in daily conditions, have a lower FT even when excluding the effect of treatment duration. Therefore, we suggest that a specific treatment for frustration intolerance may be useful in TCs. These results fit well with the “strength model” of self-regulation, which uses the metaphor of muscular exercise (and fatigue) to explain a decrease in self-control under stressful situations, leading to an “ego depletion” (extreme fatigue of self-control) (76, 77). In our study, FT would be a manifestation of self-control, which would be “depleted” in a greater extent in TC patients, due to the more severe conditions of the treatment and their lack of social support. As we propose below, future longitudinal studies could help clarify this potential inclusion of FT within the strength model of self-regulation.

Furthermore, we propose that clinical personality traits, mediated by a poor FT, may influence addiction severity, which is manifested in the type of treatment in which the patient is enrolled. Thus, a particular set of clinical personality features could be associated with a worse prognosis; however, this total effect might be better understood by the mediating role of FT. According to our results, this is the case for anxiety and PTSD personality traits. For example, a one-standard-deviation (23.8) increase in the MCMI-III anxiety scale would increase the odds to receive TC treatment by 49%. If the mediating role of FT were excluded, the association between both variables would decrease, and the odds would only increase 33.5%. In other words, a poor FT worsens the addiction prognosis of patients with higher levels of anxiety or PTSD personality traits. To our knowledge, this is the first time that the interaction between personality and FT has been explored in the context of addiction, and our results may suggest new strategies to improve withdrawal treatments. On the other hand, our conclusions are in line with classical psychotherapeutic approaches, such as rational emotive behavior therapy (78). According to this, even though a negative emotion is important to understand a behavioral outcome, implicit beliefs have a more important role in such emotion and its causes. The final goal of therapists, according to this account, is to help patients minimize anxiety, guilt, and depression by accepting themselves (79); alleviate their aggression by accepting others (80); and reduce their low FT by accepting negative events (81). Hence, frustration is considered a healthy negative emotion, but a low FT is an “irrational belief” (78). Similarly, our results suggest that an improvement in FT, as a mediational variable, may alleviate the influence of more stable personality traits on addiction severity.

Our study has a number of limitations. One aspect that should be considered is the gender composition of the samples. Females were underrepresented, and therefore, the conclusions of the study could be driven by male participants. This is a usual caveat in studies about addictions. We tackled this limitation by randomly selecting age- and sex-matched subsamples with MedCalc software. Furthermore, the size of the patient sample was relatively small. Access to a clinical sample cognitively intact and willing to participate in a research project was limited; besides that, it was challenging to complete evaluation in all cases because of treatment discontinuation. With respect to the control sample and its personality assessment, median scores are relatively high (=60) for alcohol and substance use subscales; however, in our opinion, it qualifies as a control sample for two main reasons: 1) large differences in these traits with respect to the clinical samples and 2) absence of alcohol and substance dependence and/or harm after evaluation with the AUDIT and ASSIST tools, which are more specific to detecting alcohol and substance problematic use. It should be taken into account that some of the participants in the control sample were the next of kin of those in the clinical sample. Therefore, a certain degree of similarity in the psychological variables that we assessed, such as personality traits, might occur due to an akin genetic or educational background. Considering this, the differences that were found become even more relevant. Concerning the assessment of FT, we used a semi-projective test where participants were asked to explain how they would react in a frustrating situation. It could be criticized that we did not use a task that actually induced frustration in participants. However, construct validity of Rosenzweig’s PFT has been adequately confirmed (82), it is considered a useful tool in clinical practice (83), and it has been shown to predict actual problem solving and stress coping in experimental settings (84, 85). In any case, future research should confirm our results with tasks eliciting actual frustration. Also, we did not collect information about the psychopharmacological medication that volunteers in the clinical samples were taking. Finally, it should be considered that the interplay between personality, FT, and treatment should be adequately assessed in longitudinal studies, including rates of relapse and dropouts. The conclusions of our study justify further research to elucidate this relationship.

Our study also suggests future directions to investigate the relationship between addiction, personality, and attachment. One crucial limitation is that we did not assess attachment styles in any of our samples, although our results can be interpreted to draw some preliminary conclusions and inspire future research. First of all, previous results show the relationship between a disorganized attachment and a higher probability of suffering anxiety (86), depression (87), addiction (88), or PTSD (89), and expressing personality disorders such as borderline, avoidant, or antisocial, among others (90). In turn, recent research has shown an association between insecure attachment, alcohol/substance addiction, and an increased amount of borderline personality organization (57). Even though the borderline trait did not survive Bonferroni correction in our study, it was suggestive of symptoms at a subclinical level in the clinical samples included in our study. Moreover, the relationship between attachment styles and substance addiction has been proven to have a biological correlate, in particular, white matter integrity: in a sample of poly-drug users, Unterrainer et al. (91) showed a decreased fractional anisotropy compared with recreational users or non-users. Furthermore, impairment of the superior longitudinal fasciculus and corona radiata was associated with an insecure attachment and negative affectivity. Interestingly, this research group previously found a relationship between white matter integrity, attachment styles, and personality factors in the aforementioned tracts (92): in this case, structural connectivity impairment positively correlated with anxious attachment and personality dysfunctioning, whereas white matter integrity positively correlated with openness and agreeableness.

Early attachment relationships, which are based on the mental models that children build about themselves, their interrelationships with their caregivers, and their environment, are essential for them to acquire the abilities of emotional management, attentional control, mentalizing, and autonomy (93). In turn, attachment theory is becoming strongly influential in research and intervention on personality disorders (94–96). According to Adshead and Sarkar (96), these disorders include an intrapersonal component (related to a dysregulation of arousal, impulse, and affect systems in response to stress), an interpersonal component (dysfunctional attachment patterns), and a social component (dysfunction in social behaviors). To some extent, our research covers an interpersonal (FT) and social component (substance addiction inpatient or outpatient treatment), which can be related with the interpersonal component. According to our results, anxiety and PTSD are those personality traits more affected in the TC group, and under the influence of FT, they predict the inclusion in TC or AT treatment. The link between attachment styles and anxiety has been extensively demonstrated [see, for example, the review by Ref. (97)]. According to these authors, anxiety is more frequent in adolescents who experienced resistant attachment during childhood compared to those with secure or avoidant styles. This association remained when considering attachment-related negative experiences during childhood (such as parental divorce or loss) or attachment states of mind (i.e., preoccupied), instead of self-reported assessment of attachment. Similarly, PTSD has been related to disorganized attachment. For instance, unresolved attachment-related state of mind is associated with a higher risk (7.5) of expressing PTSD (98). Furthermore, posttraumatic symptoms through midlife and old age are associated with adult attachment insecurity (99). In conclusion, our results point to a plausible interplay between disorganized attachment, FT, and certain personality traits (mainly anxiety and PTSD) in substance abuse disorders. Future research on these topics from a unitary perspective may increase our understanding of substance addiction, improving prevention policies, and hopefully designing improved individualized treatments for patients suffering this devastating disorder.

## Ethics Statement

This study was approved by the Committee of Ethics in Research of the University of Navarra. All subjects gave written informed consent in accordance with the Declaration of Helsinki.

## Author Contributions

FG, JB, and IM conceived the study; DR-C, CG-R, FG, and IM collected data and organized the database; DR-C, IM, and JB performed the statistical analyses; JF-M wrote sections of the manuscript and supervised data analyses. All authors contributed to manuscript revision, read and approved the submitted version.

## Conflict of Interest Statement

The authors declare that the research was conducted in the absence of any commercial or financial relationships that could be construed as a potential conflict of interest.
